# Discovering Study-Specific Gene Regulatory Networks

**DOI:** 10.1371/journal.pone.0106524

**Published:** 2014-09-05

**Authors:** Valeria Bo, Tanya Curtis, Artem Lysenko, Mansoor Saqi, Stephen Swift, Allan Tucker

**Affiliations:** 1 Department of Information System and Computing, Brunel University, London, United Kingdom; 2 Rothamsted Research, Harpenden, United Kingdom; Leibniz-Institute for Farm Animal Biology (FBN), Germany

## Abstract

Microarrays are commonly used in biology because of their ability to simultaneously measure thousands of genes under different conditions. Due to their structure, typically containing a high amount of variables but far fewer samples, scalable network analysis techniques are often employed. In particular, consensus approaches have been recently used that combine multiple microarray studies in order to find networks that are more robust. The purpose of this paper, however, is to combine multiple microarray studies to automatically identify subnetworks that are distinctive to specific experimental conditions rather than common to them all. To better understand key regulatory mechanisms and how they change under different conditions, we derive *unique networks* from multiple independent networks built using *glasso* which goes beyond standard correlations. This involves calculating cluster prediction accuracies to detect the most predictive genes for a specific set of conditions. We differentiate between accuracies calculated using cross-validation within a selected cluster of studies (the intra prediction accuracy) and those calculated on a set of independent studies belonging to different study clusters (inter prediction accuracy). Finally, we compare our method's results to related state-of-the art techniques. We explore how the proposed pipeline performs on both synthetic data and real data (wheat and Fusarium). Our results show that subnetworks can be identified reliably that are specific to subsets of studies and that these networks reflect key mechanisms that are fundamental to the experimental conditions in each of those subsets.

## Introduction

All organisms have many mechanisms, necessary for their survival, that carry on mostly unchanged under all conditions that the organism is subjected to (e.g. cell metabolism). Other mechanisms, however, occur only when some event external or internal to the organism (environmental changes, stress, cancer, etc.) happens and triggers them. Some conditions might trigger similar mechanisms (more or less based on how similar the conditions are) that researchers identify using consensus networks analysis that identifies links in common over a number of studies [1]. Highlighting the similarities, though, can overshadow or even hide what is unique and typical to one specific condition. Biologists are clearly interested in what these similarities are but they are also interested in identifying the condition-specific mechanisms/gene-paths of which knowledge will help in their detailed understanding. The novelty of our approach is the ability to semi-automatically identify subnetworks that are unique to a number of independent studies (unique networks). Identification of unique networks could lead to a better understanding of those mechanisms.

In this paper we extend the work presented in [2] by formally deriving a *unique network*, exploring the results on different simulated datasets to gain a better understanding of the performances of our pipeline in worst/best case scenarios. We also compare *glasso* with Weighted Gene Correlation Network Analysis (WGCNA) for the identification of the unique networks. The approach is also applied to another real microarray dataset (*Fusarium*) and we further explore the biological validation of the wheat results. We choose to focus on wheat because knowledge of it is still very sparse and understanding its gene regulation is challenging due to the size and complexity of its genome.

Microarray data measure the simultaneous expression of thousands of genes allowing the modelling of the underlying mechanisms of gene regulation through Gene Regulatory Networks (GRNs). Because of the structure of microarrays (thousands of genes vs tens of samples) the integration of these data, collected from different studies, is an ongoing problem with some reported successes [3]. In [4] the authors present a method for the unsupervised integrative modelling of multiple datasets which models each dataset using a multinomial Dirichlet allocation mixture model and captures the underlying structural similarities between them. In [5] more robust models are built from multiple datasets by ordering them based on the level of noise and informativeness and using different Bayesian classifiers to select the informative genes. Steele et al. [6] combine various microarray datasets using post-learning aggregation to build robust regulatory networks. We adopt a similar approach in this study, but rather than generating the *consensus* of all datasets we identify mechanisms that are *specific* to a subset of studies. [1] also explore consensus approaches but use a clustering technique coupled with a statistically based gene functional analysis for the identification of novel genes. Often the sheer number of genes makes the understanding of GRNs difficult and sometimes modules are created by grouping genes that perform some similar function [7]. Networks of these modules can then be discovered to identify mechanisms at a more general level. Clustering helps to preserve all information but might increase bias. In [8] two cancer datasets are compared (case and control). For each dataset, pairwise correlation of gene expression profile is computed and then used to build a frequency table. The values in the table are then used to build a weighted gene co-expression frequency network. After this they identify sub-networks with similar members and iteratively merge them together to generate the final network for both cancer and healthy tissue. Alaakwaa et al. [9] instead explore the biclustering technique [10] which aims to cluster both genes and samples simultaneously. They apply six different biclustering methods and use the resulting biclusters to build Bayesian Networks for each and finally merge the results in one single network which captures the overall mechanism.

In this paper, rather than focusing on consensus networks, we develop a method to identify, given a set of different studies (clusters of studies), the differences between each other and particularly what makes each study unique compared with the others in the input set. Therefore, we explore a method to 'home in' on the differences of GRNs generated from different studies by using a combination of clustering, network discovery and graph theory. We go beyond the simple pairwise correlations between genes which is common in many studies e.g. [8] by building independent networks for each study using *glasso* which identifies the inverse covariance matrix using the lasso penalty to make it as sparse as possible. Then, we cluster the studies with similar regulatory behaviour (similar network structure) using an adaptation of the sensitivity formula as a graph similarity measure. In order to avoid any confusion from now on, we refer to each cluster of studies as a ‘*study-cluster*’. At this point we detect the edges that are unique/specific for each *study cluster*. Furthermore, we use these results to build Bayesian Networks for each study-cluster to identify the most predictive group of genes and further refine our unique networks.

As a validation of our pipeline we compare the *glasso* technique with Weighted Gene Correlation Network Analysis (WGCNA).

We also exploit, as a further comparison to our entire method, a popular state-of-the-art technique known as Biclustering, which aims to simultaneously cluster genes and samples, but not at the network level.

To investigate different organisms and demonstrate the generalization of the approach to different microarray data, we explore the performances of the pipeline, first, using a simulated dataset as input and then using wheat and *Fusarium* datasets.

## Materials and Methods

The pipeline described here, which we call UNIP (Unique Network Identification Pipeline) aims to discover what genes and the relationships between them are specific to the study or group of studies under consideration. To achieve this goal, we, first, identify the variables/genes that uniquely appear in the GRN of one study or one group of studies, and then derive study-specific gene regulatory networks (*unique networks*). *Unique networks* can be seen as the sub-GRNs specific to the group of studies. This helps biologists to identify what are the typical mechanisms that characterize one study rather than another.

**Figure 1:**
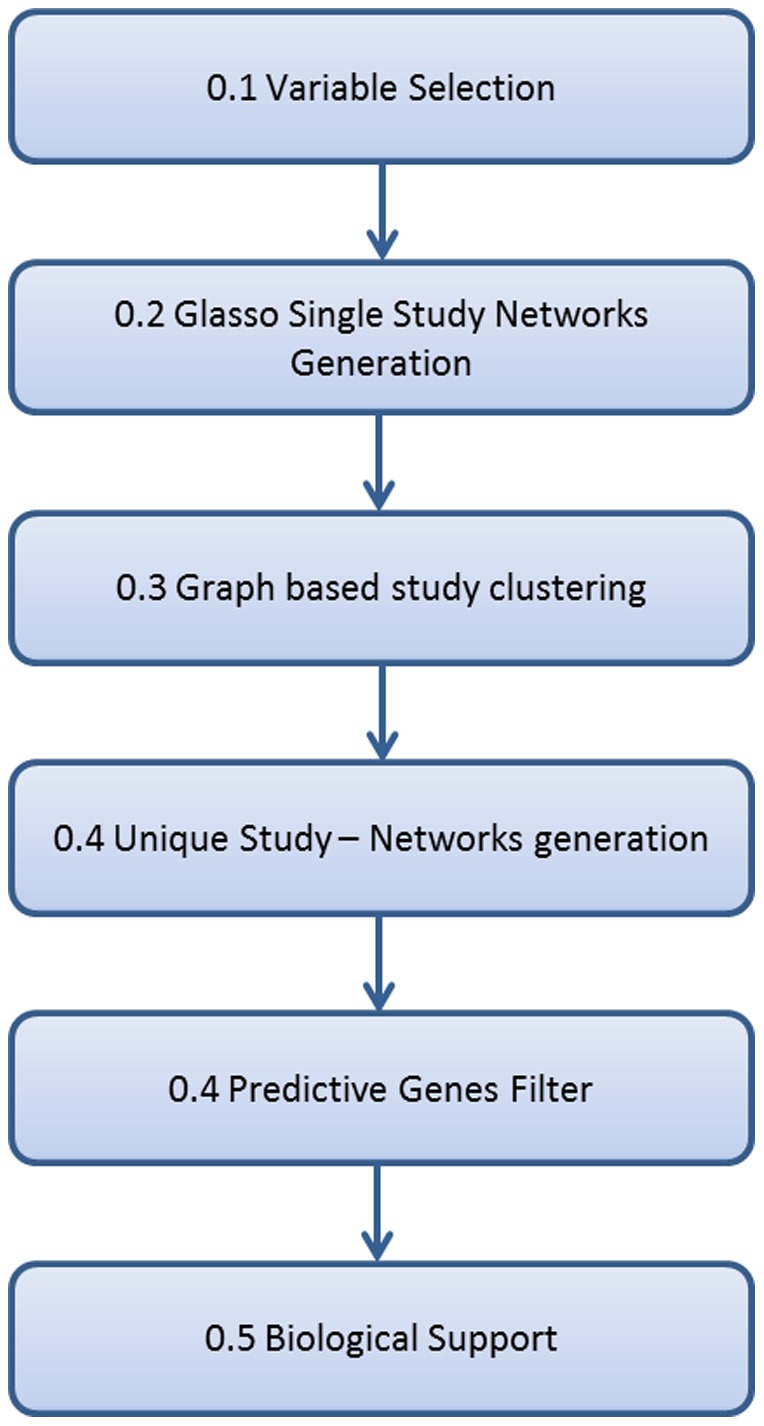
Flowchart of the steps for the pipeline. The figure shows the main steps that constitute the pipeline. Each step is properly described in this section.

To achieve this we need to sequentially go through a list of steps, each with a specific purpose. Figure 1 represents the flowchart of the steps involved, each explained in the following sections.

### 0.1 Selection of Informative Genes

A key characteristic of microarrays is the simultaneous measurement of a large number of genes in the order of thousands and a less numerous amount of samples (in the order of tens or hundreds). This allows biologists and bioinformaticians to have a general view of the behaviour of the organism. Although it is not our focus, for computational and practical reasons we need, first, to reduce the number of the variables involved. To prevent noise and bias we choose to avoid clustering and simply discard all non-informative genes. Firstly, we discard those genes that are currently not in the Gene Ontology (GO) [11] database, meaning we can focus on genes that we can validate biologically. We then consider the expression profiles' standard deviation of the genes within the single studies and reject those with a standard deviation lower than a threshold set to 2. At this point to further improve the overall information quality, as we are integrating data across studies, we select only those genes that survived in at least 25% of the studies.

### 0.2 Glasso

Each organism has underlying mechanisms which apply under normal conditions. When the same organism is subjected to different conditions (stress, environmental changes, etc.) then it will need to respond to the change resulting in new paths of genes being highlighted. This results in new underlying mechanisms and/or changes in already active mechanisms. So, different experimental conditions can show different Gene Regulatory Networks (GRNs).

Once we have selected the most informative genes, we need to build a (GRN) for each condition/study in the dataset.

GRNs have two main components: nodes which represent the variables/genes and edges that encode the joint probability distribution by representing conditional independences between variables. As we want to identify networks that go beyond simple pairwise relationships, our procedure uses *glasso*, an algorithm that scales well for a large number of variables/genes.

The problem of identifying the structure of the network can be solved by estimating the relationships between variables. In the case of undirected graphs it is the same as learning the structure of the conditional independence graph (CIG), which in the case of Gaussian random variables, means to identify the zeros of the inverse covariance matrix (also called a precision or concentration matrix). Given a *p*-dimensional normally distributed random variable *X*, assuming that the covariance matrix is non-singular, the conditional independence structure of the distribution can be represented by the graphical model G = (N, E) where N = (1,..,*p*) is the set of nodes and E is the set of edges in N
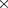
N. If a pair of variables is not in the set E it means that the two variables are conditionally independent given the other variables. This corresponds to a zero in the inverse covariance matrix. Therefore it imposes an 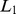 penalty in the estimation of the inverse covariance matrix in order to increase its sparsity [12], [13].

The *glasso* package in R estimates a sparse inverse covariance matrix using a lasso (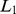) penalty. Suppose, we have N multivariate normal observations of dimension p, with mean 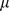 and covariance 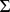. The problem is to maximize the penalized log likelihood 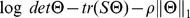 where 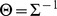, *S* is the empirical covariance matrix and 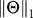
is the 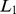 norm the sum of the absolute values of the elements of 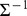
and 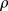 is the regularization parameter. The parameter 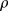 can be a scalar (typical situation) or a p
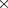
p matrix. If 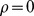 means no regularization [12]. In this paper we apply the *glasso* package, with 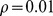, to build one network for every study in the microarray dataset.

### 0.3 Graph Similarity

We integrate several microarray datasets in order to compare different studies. Some studies will still have some network paths in common (if the genes are regulating one another under those conditions). For example, heat stress and drought stress will have gene pathways in common with other stress-related studies. So, at this point of our pipeline the objective is to automatically detect mechanisms common to similar studies and cluster them using an adaptation of the sensitivity metric to obtain a restricted number of *study-clusters*. Given two networks, network 1 (N1) and network 2 (N2), the connections that two networks have in common are the true positives, those that are in N1 but not in N2 are the false positives and those not in N1 but in N2 are the false negatives. Therefore, we analyse the connections in common between two study-networks and build a contingency table. To verify the reliability of the clusters we compare the results with the description of the studies available when downloaded from public databases such as ArrayExpress [14]. We explored a number of clustering techniques but found that k-means (R function based on [15]) generated the most convincing study-clusters. In the process of identifying unique networks we first build the consensus network (which identifies the network pathways that are common to a certain groups of networks) [6] for each study-cluster as a representative of the general mechanism for that group of studies. We select only those edges that exist in the consensus-study network in consideration, but not in the other consensus-studies networks. The resulting list of nodes involved in the unique connections are used to build the unique Bayesian networks as explained in detail in the next section.

### 0.4 Unique Bayesian Networks and Prediction

In the step described above we cluster the studies to group them, if possible, in k (k-means parameter which allows us to establish the number of clusters needed) generic conditions. For each study-cluster a consensus network is constructed, that represents the underlying gene regulatory mechanism(s) in common for that group of studies. This will allow us to build more robust GRNs for each study-cluster. As explored in the Introduction, consensus networks as consensus clustering is a popular approach but in [2] we introduced and now we explore further what we call *unique networks*.

Given a generic graph 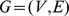. We have *m* fixed graphs 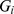 such that 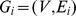, where 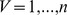 is the set of vertices(nodes) of the graph and 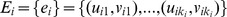, 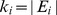 and 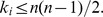 We define the unique function as 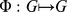, where, given 
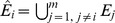


**Definition 1**: We define a function 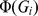 such that 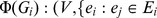 and 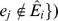


The unique function returns what we call a unique network. It consists of the same set of edges in the consensus network in consideration except those that also exist in the remaining consensus networks.

We choose to validate the networks through prediction using Bayesian Networks (BNs) which naturally perform this using inference, given the graphical structure obtained using the gene in the unique networks provided by *glasso*. BNs [16], [17] are a class of graphical models that represent the probabilistic dependencies between a given set of random variables. A Bayesian network has a set of variables called nodes and a set of directed edges between variables called arcs. The nodes and arcs together form a *directed acyclic graph (DAG) G = (V,A)*. Each variable in the network has attached a conditional probability table of itself given the parents. Having reduced the number of variables and samples by identifying the unique networks, we build one BN for each of the study-clusters previously identified based on the genes with unique edges in the *glasso*-derived networks. To do this we used the R package *bnlearn* [18], [19]. After this we are interested in finding the most predictive (how well it predicts other expression level values) and predictable (how well its expression level values are predicted) genes within (intra) and outside (inter) the study-clusters using the leave one out cross validation technique. The idea is that genes that are predictive or predicted better within the selected study-cluster than on other studies are more likely to be relevant to the unique network. Given the *m* studies and *n* genes within each studies-cluster we use *m*-1 studies as a training set and the remaining one as test set. Then we employ the R package *gRain* [20] which, given the *n*-1 genes, predicts the expression value of the one left out. We compare the predicted value of the left out gene with its real value, return 1 if they correspond and zero otherwise. We do this within all the study-clusters and for all possible combinations of training and test sets of studies and genes. Finally, we average the amount of correctly-predicted values among the total predictions to obtain the *correct-prediction* for each gene.

### 0.5 Biological Support

Having identified the study-clusters and, in turn, the study-specific mechanisms within the *unique-networks*, we explore the biological meaning behind them. To do this, we exploit two pieces of software:

1. *Mapman* [21] which explores gene-by-gene the functions related to it and returns a list of functions and a graph of connections

2. The AIC-MICA method [22]. The method identifies functions in the biological process aspects of the Gene Ontology that best characterise particular groups of genes. It uses both the structure of the ontology and a term specificity measure (information content, IC) to find terms that are both biologically specific (e.g. not too high-level) and applicable to the largest possible subset of each group. Therefore, unlike the over-representation measures, it gives a general idea about the role of the cluster as a whole and a level of ontology at which such commonality could be found (e.g. average IC of the found terms).

The combination of these tools allows us to identify gene functions that are characteristic of the study-cluster in consideration, adding credence to our findings.

Finally, in the case of the wheat dataset, to prove that the results are robust and consistent, we conduct a search in the literature for every gene involved in the *unique-networks* and its connections. The results of this research are explained in the Discussion session.

**Figure 2:**
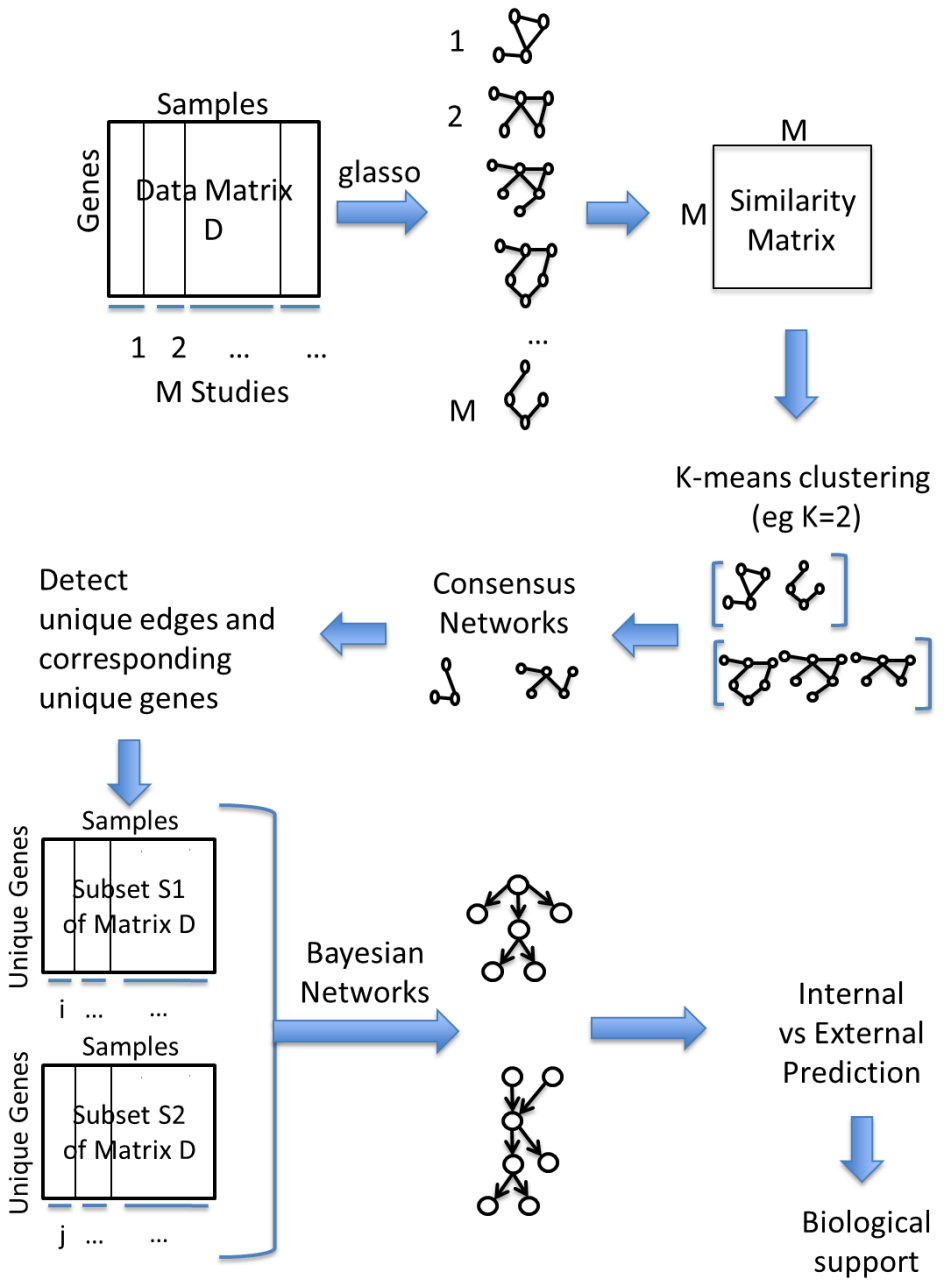
Pipeline overview. A schematic overview of the sequence of steps forming the pipeline.

An overview of the pipeline is shown in Figure 2.

### 0.6 WGCNA

One of the main goals of this paper is to explore techniques that go beyond simple pairwise correlations. Thus, we first explored the *glasso* algorithm described above and then we compare the results using a co-expression network analysis technique known as Weighted Gene Correlation Network Analysis *WGCNA* [23]–[25]. WGCNA uses a thresholding procedure raising the co-expression similarity to a power: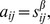 with 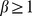 to transform the co-expression similarity matrix into the adjacency matrix. To pick the right value of beta WGCNA uses a biologically motivated criterion referred to as the scale-free topology criterion [26], [27], as opposed to the random graph model [28].

### 0.7 Biclustering

One of our pipeline's purposes is to identify groups of genes that are important for a specific set of conditions. Therefore, we compare our results (the discovered clusters and their associated networks) with *Biclustering* techniques which aim to cluster samples and genes simultaneously [10]. It is important to highlight that biclustering works on each sample and not on the studies. There are various implementation variants in the literature for biclustering [29] but for this work we specifically chose a method called Questmotif which is based on the framework described in [30], for the simulated (categorical) datasets and the BCS method for the real datasets of wheat and *Fusarium*. BCS is a state-of-the-art method that normalizes the data matrix and looks for checkerboard structures using the well-known technique of singular value decomposition in eigenvectors applied to both rows and columns [31]. Both BCS and Questmotif are implemented in the R package *biclust* [32].

## Results

We first apply our pipeline to a well-known and easily modifiable dataset in order to measure and test the performance of our method. Once we verified its applicability we explore wheat and Fusarium microarray dataset. A schematic flowchart of the steps applied to all three datasets is shown in Figure 1. This involves firstly selecting the variables by applying a standard deviation threshold, followed by the generation of single study networks using *glasso* and clustering of the studies using a graph similarity analysis that returns the *unique networks*. Finally a further filtering of the genes based on their prediction value and the validation of the results with biological feedback.

### Results on simulated data

First of all we evaluate our pipeline's performance using simulated data whose characteristics are well defined. [33] is a synthetic Bayesian networks repository database from which we select three networks. We select the networks: Alarm [34], Insurance [35] and Child [36] with 37, 27 and 20 nodes respectively. For each network we download the structure and the associated dataset and take 200 samples. At this point we have three datasets of sizes: 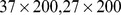 and 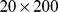. Each dataset is representative of a different underlying structure (much like a gene network under different experimental conditions).

In this dataset we assume that the 84 total variables are already the results of the variable selection described in Section 0.1 since this is not the main focus of our work.

**Figure 3:**
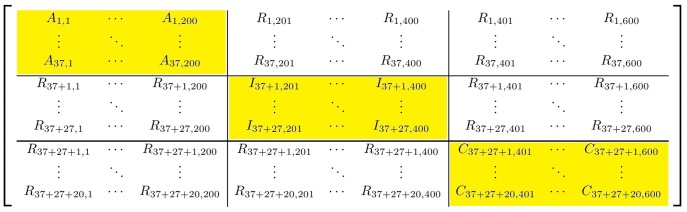
*Big matrix* constructed from the datasets generated from the three networks and six randomly generated datasets which represent the noise. The shaded regions indicate the non-noisy datasets generated from Alarm, Insurance and Child networks (respectively A, I and C in the figure).

To test our pipeline, we mix the datasets adding noise and create what we will call from now on *big matrix*. *Big matrix* is composed of 9 smaller matrices. Three matrices are the datasets sampled from the networks while the remaining six are randomly created based on the values of the original variables/nodes. If we consider the *big matrix* as a 3×3 block matrix composed of nine blocks, each row of the *big matrix* has one sampled dataset and two random ones. Figure 3 shows a representation of the *big matrix* where the capital letters A, I, and C indicate the datasets of Alarm, Insurance and Child respectively while R represents random values (noise).

We gather the 600 samples in 15 studies of 40 samples each so that each column-block of *big matrix* contains exactly 5 studies of 40 samples each and 84 variables/nodes (37+27+20). In Table 1 we show the correspondence of studies and original networks.

**Table 1:** Simulation studies generated independently from the three networks in consideration.

Simulation Studies	
Studies	Network
1,2,3,4,5	Alarm
6,7,8,9,10	Insurance
11,12,13,14,15	Child

In order to test the robustness of our pipeline we gradually introduce noise by swapping actual samples with random values. We first analyse the *big matrix* with no noise (0%). Then, we gradually introduce an increasing percentage of random samples of the total (noise) and decide to focus on what we find to be the most revealing noise-levels: 10%, 50% and 90%.

**Figure 4:**
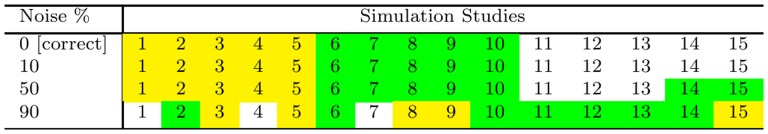
Study-clusters for the original data (0% of noise), 10%, 50% and 90% of noise. The studies' number highlighted with the same colour belong to the same cluster.

Once we have selected the relevant variables (see Methods and Materials) we create one network for each of the 15 studies. Due to the categorical nature of the data, we decided to use bayesian networks rather than *glasso* with a simple hill climbing approach [37]. Ideally, we want the pipeline to cluster the studies as they belong to the original networks and to detect for each study-cluster the variables that are truly involved. We use a simple k-means [15] approach to cluster the networks based upon a graph similarity metric (see Materials and Methods section). Figure 4 shows the clusters' arrangement for the original data and for the data with an increasing amount of noise (from 10% till 90%). While at 10% of the noise the study-groups detected by our pipeline reflect the real studies arrangement, an increase to 50% disrupts the process and shuffles the studies. As expected, the noisier the input is, the more mixed the study-groups are. We want to see how robust our unique network pipeline is to this level of noise.

The next step in the pipeline is to compare each original network with the others based upon their cluster assignment. For each cluster of networks we build both consensus (where links in the network must exist in all networks for that cluster) and unique networks (where links must only occur in that cluster): steps 1 to 4 of our pipeline (see Figure 1). *Big matrix* contains all 84 variables from all the three networks, which leads to the fact that all the unique study-cluster networks will most probably include variables and connections that do not belong to the original structure.

**Figure 5:**
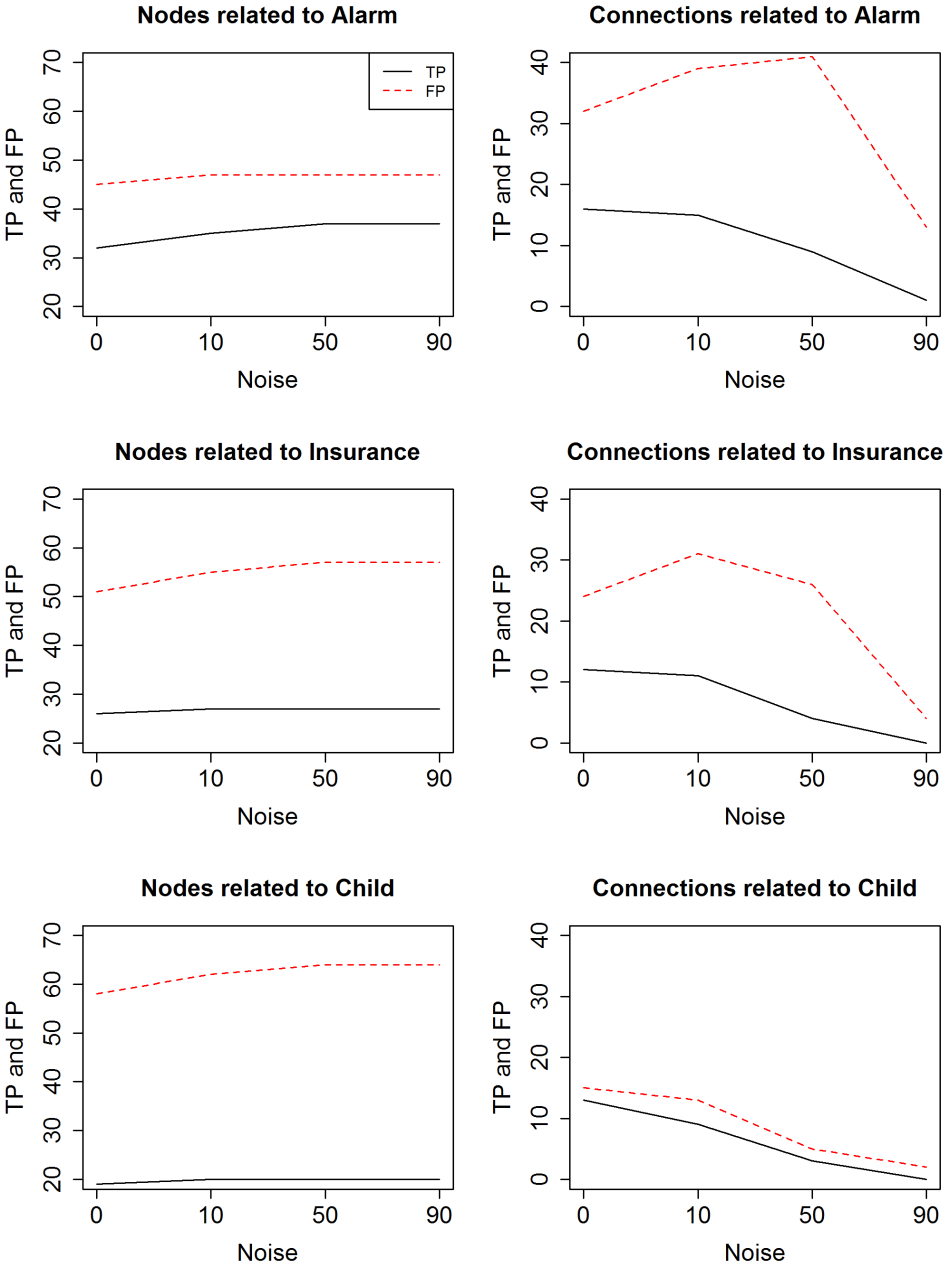
TPs and FPs vs noise before calculating the correct-prediction. The figures show the evolution of TPs and FPs vs noise in terms of nodes (variables involved in the discovered subnetworks) and connections between nodes. These are the partial results, prior to the filtering of the informative nodes based on the intra cluster correct-prediction accuracy (which are shown in Figure 6).

Figure 5 shows these first intermediate results for detecting the true positive (TP) nodes and connections between nodes as noise increases. TPs are the number of connections/nodes in the simulated network that are also in the original network. This corresponds to the step *0.4 Unique study-networks* in the pipeline flowchart, before non-predictive variables are filtered out. The number of both TPs and FPs nodes for all the clusters only slightly increase along with noise. This is due to the fact that at zero noise the pipeline manages to already select the majority of the correct nodes.

The connections, on the other hand, behave differently. For lower percentages of noise in (Alarm and Insurance) FPs tend to increase very slightly. When the data becomes almost completely random, the algorithm recognizes the faulty information and massively decreases the number of connections detected to zero. One way to decrease the number of FPs, especially for the nodes, would be to increase the number of samples per study in the input dataset. Some tests proved that samples need to be more than 200 which is not possible for microarray datasets.

To summarize, at this stage of the pipeline we discovered that for low levels of noise our pipeline can robustly identify unique networks and it is also resilient to moderate noise. High levels of noise, however, appear to affect the TPs and FPs of the connection identification more than the node identification.

Finally, we calculate the 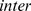 and 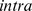 clusters prediction to validate the predictive power of the subnetworks for datasets that are clustered together and to filter out any nodes that do not appear to be uniquely predictive to their study-group.

**Figure 6:**
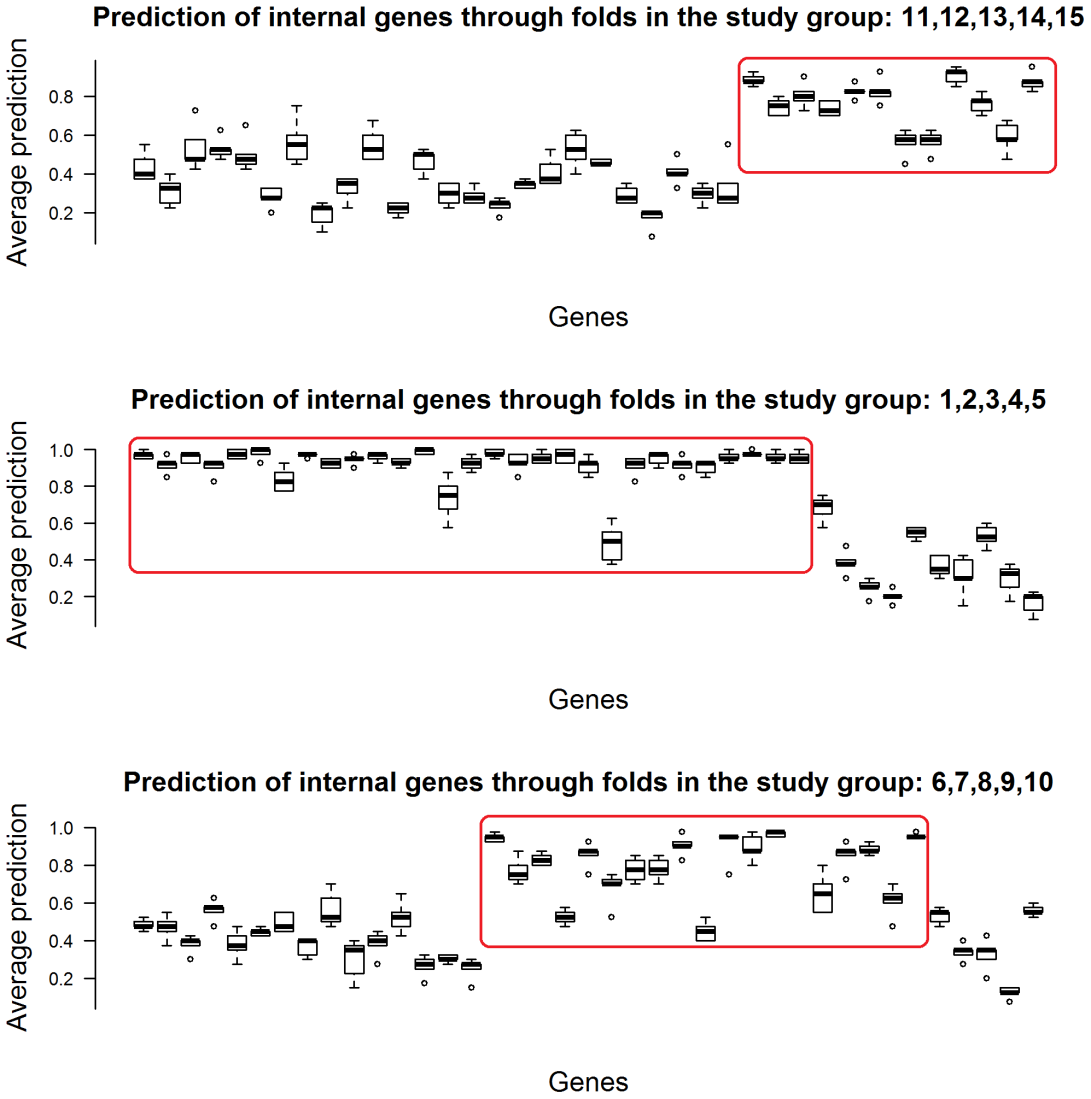
Intra cluster correct-prediction for simulated data. The figure shows the boxplots of the intra cluster correct-prediction (calculated within the same cluster using cross-validation) for the simulated dataset in the case of 0% of noise.

The possible states of the variables vary from 2 to 6. As a result, the chance to correctly predict them varies from 0.5 to 0.2. The variables in the alarm networks are categorical with a maximum of 4 possible states. Out of 37 variables, 13 have only two possible states, 17 have 3 possible states and only 7 have 4 possible states. So, to be able to say that one variable is predicting better than chance, its average correct-prediction across training and test sets has to be higher than its *accuracy by chance*. The graphs in Figure 6 represent (in the case of 0% noise) the boxplot of the average *correct-prediction* across training and test within each of the three study-groups, including all the variables involved in the unique network for that group. The study-clusters are listed in the titles and we can refer to table 1 to identify the networks they belong to. The variables involved in the unique networks for each group of studies are listed in the x axis. We clearly see groups of variables that stand out. The variables that truly belong to the corresponding real networks result in having an average accuracy above 0.6 which is significantly higher than their *accuracy by chance*. The circled variables are the ones with the highest correct-prediction and are likely to be the ones that are involved in the original networks.

**Figure 7:**
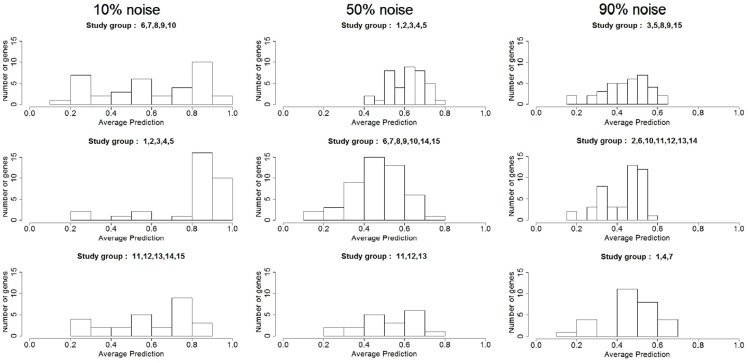
Intra cluster correct-prediction distribution for 10, 50 and 90% perturbation. The figures show the histograms of the intra cluster correct-prediction (calculated within the same cluster using cross-validation) for the simulated dataset for different levels of noise.

**Figure 8:**
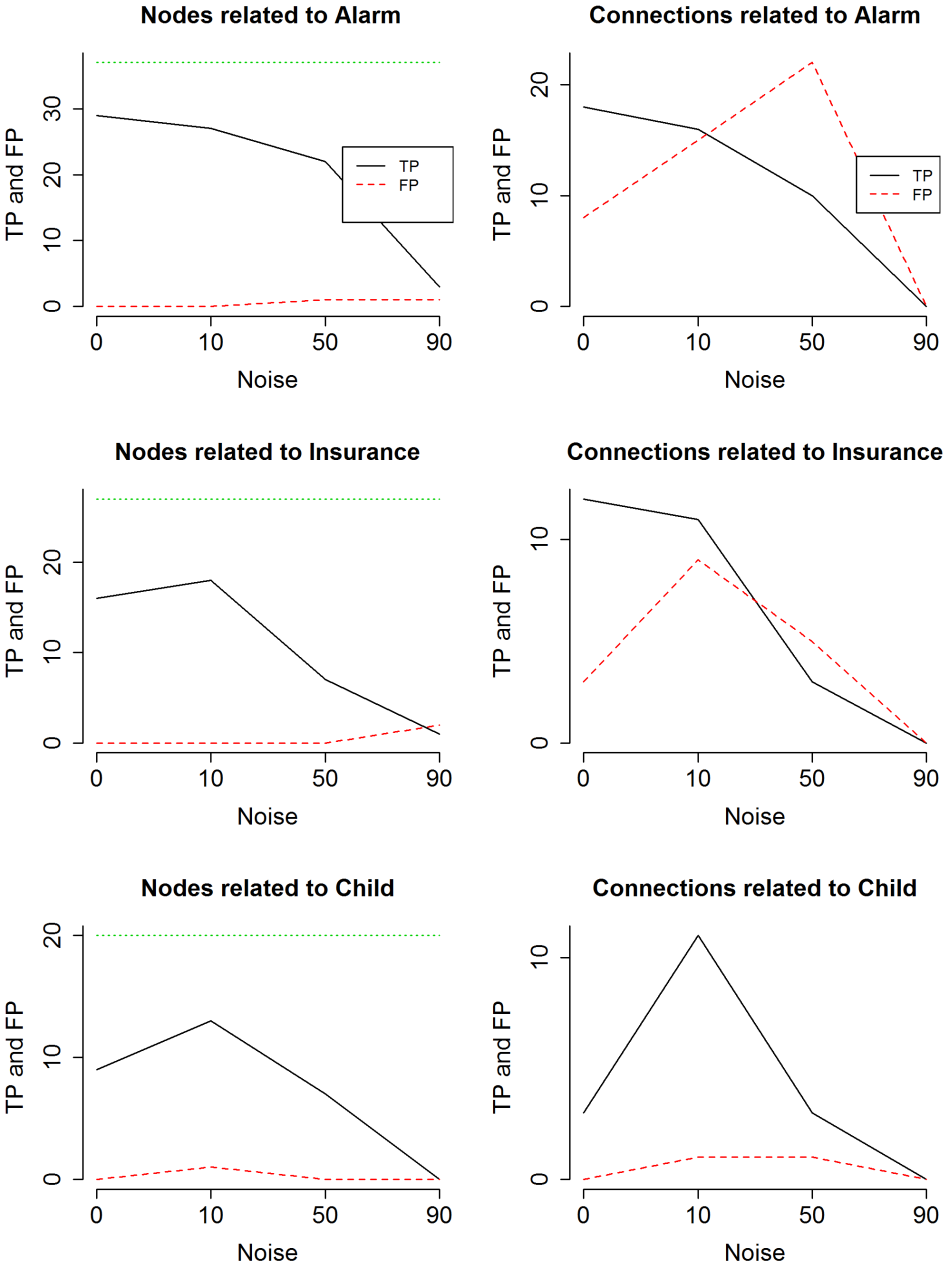
TPs and FPs vs noise after calculating correct-prediction. The graphs show the number of TPs and FPs nodes and connections detected at different levels of noise. Threshold set to 0.6. The dotted lines at the top of the graphs indicates the number of nodes in the relative original network.

Similarly, Figure 7 shows the distribution of the node's intra cluster correct-prediction when the noise is increased to 10, 50 and 90%. As we increase the noise, a number of things come to our attention. For lower percentages of noise, the variables' accuracy histogram shows one major peak at high correct-prediction values and, in the first and third graph of Figure 7, another smaller peak at low correct-prediction values creating bimodal distribution. While the higher peak indicates the TPs, the lower one identifies the amount of FPs. An increase of noise, however, gives a more uniform distribution. Even for the highest level of noise there are still a good number of nodes with relatively high intra cluster (within the same *study-cluster*) correct-prediction levels. This gives us confidence that even for the noisiest datasets, the pipeline is still capable of identifying key variables. Following the flowchart, we now select the variables that truly are involved in the network mechanism setting a threshold for the accuracy (*0.4 Predictive genes*). Different thresholds return a different number of TPs and FPs. Results show that for a threshold accuracy of **0.6** we obtain the best combination of TPs act while the number of TPs is very high, the number of FPs is reduced to zero. Which means that calculating the intra cluster correct-prediction allows to discard all the variables that are not involved in the original network. Figure 8 shows the behaviour of FPs and TPs as the noise increases.

As expected, when we increase the noise TPs' trend decreases while FPs slightly increases. The noisier the data are, the more difficult it is to set a threshold for the variables. The reasons for this are twofold: because the trend of FPs is higher and because both trends reach zero very quickly. Even if the number of TPs detected by the pipeline decreases when the noise level exceed 0.5, the number of FPs remains close to zero for all level of noise. This shows that even for extremely noisy and biased input data, the pipeline is still able to detect variables that are highly important.

### Biclustering

We now compare our pipeline with a biclustering method called Questmotif which is based on the framework described in [30]. Biclustering identifies both genes and samples simultaneously so whilst subnetworks are not discovered (which our approach focusses on), it should at least identify variables that are clustered for specific studies. We apply biclustering to the same *big matrix* dataset of 600 samples and 84 variables, and exploit the results. Questmotif detects 9 biclusters. Cluster 1 groups 124 samples out of which 122 belongs to network alarm, and 8 variables all involved in the alarm mechanism. Cluster two groups 261 samples of which 190 belongs to network insurance and only two genes both belonging to the insurance network. Cluster 3 groups 93 samples, 88 of which belong to the child network along with 4 variables from the child network. Bicluster 4 groups 20 samples and 10 variables from the alarm network. Bicluster 5 still groups a majority of samples belonging to alarm. The remaining clusters groups have mixed samples and mixed variables in a very low number. Overall, bicluster does not perform as well as our pipeline. It manages to identify a respectable number of correct samples, but fails at detecting as many corresponding true variables as our pipeline (and no connections are discovered as it is not a network-based approach).

In conclusion, the simulated data study indicates that our pipeline works extremely well for clean data and is reasonably resilient to noise until 50% of the data is affected. Both the network clustering process and the detection of variables that truly belong to the original networks seem robust and only fail at higher level of noise. In the following section we will use our method with two sets of real microarray data studies: Wheat and *Fusarium*. Based on the results, wheat datasets behave similarly to the case of zero or very low noise, while *Fusarium* appears to be associated with noisier data as a result of more clearly defined conditions for wheat.

### Wheat microarray data

We now focus on the analysis of various wheat transcriptome datasets derived from multiple experiments of plants subjected to a range of treatments: stress, development, etc. Unprocessed wheat microarray expression data for this work was downloaded from ArrayExpress database [14]. Only studies using GeneChip Affymetrix Wheat Genome Array technology that profiled wheat species were included. The combined dataset was pre-processed using Robust Multichip Average method [38] and redundancy-adjusted Pearson correlation coefficient was calculated according to the method described in [39]. We analyse a microarray dataset of 61290 genes common to 523 samples, grouped in 16 studies. Each study represents a different treatment the plant has been subjected to, as shown in Table 2. Each study contains samples derived both from treated and non-treated samples. Studies 1–6, 12, and 13 are considered stress-enriched, and the remaining as non-stressed treatments. Labels are taken from [40].

**Table 2:** Study numbers, labels, number of samples and descriptions of the wheat microarray dataset.

Wheat Studies			
Study	Label	Number samples	Description
1	E-MEXP-971	60	Salt stress
2	E-MEXP-1415	36	S and N deficient conditions
3	E-MEXP-1193	32	Heat and Drought Stress
4	E-MEXP-1694	6	Re-supply of sulfate
5	E-MEXP-1523	30	Heat stress
6	E-MEXP-1669	72	Different nitrogen fertiliser levels
7	E-GEOD-4929	4	Study parental genotypes 2
8	E-GEOD-4935	78	Study 39 genotypes 2
9	E-GEOD-6027	21	Meiosis and microsporogenesis in hexaploid bread wheat
10	E-GEOD-9767	16	Genotypic differences in water soluble carbohydrate metabolism
11	E-GEOD-12508	39	Wheat development
12	E-GEOD-12936	12	Effect of silicon
13	E-GEOD-11774	42	Cold treatment
14	E-GEOD-5937	4	Parental genotypes 2 biological replicates from SB location
15	E-GEOD-5939	72	36 genotypes 2 biological replicates from SB location
16	E-GEOD-5942	76	Parental and progenies from SB location

**Figure 9:**
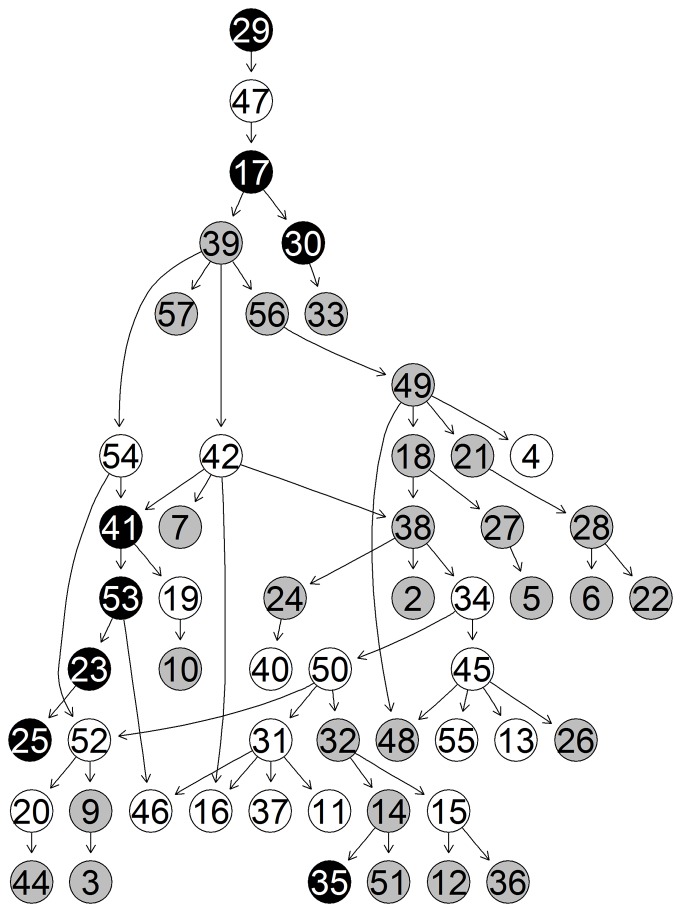
Network 1. Unique-Network for wheat under stress-enriched conditions in cluster 1. Grey nodes indicate highly predictive (average correct-prediction level higher or equal to 0.6) genes. Black nodes highlight highly predictive and stress related genes.

**Figure 10:**
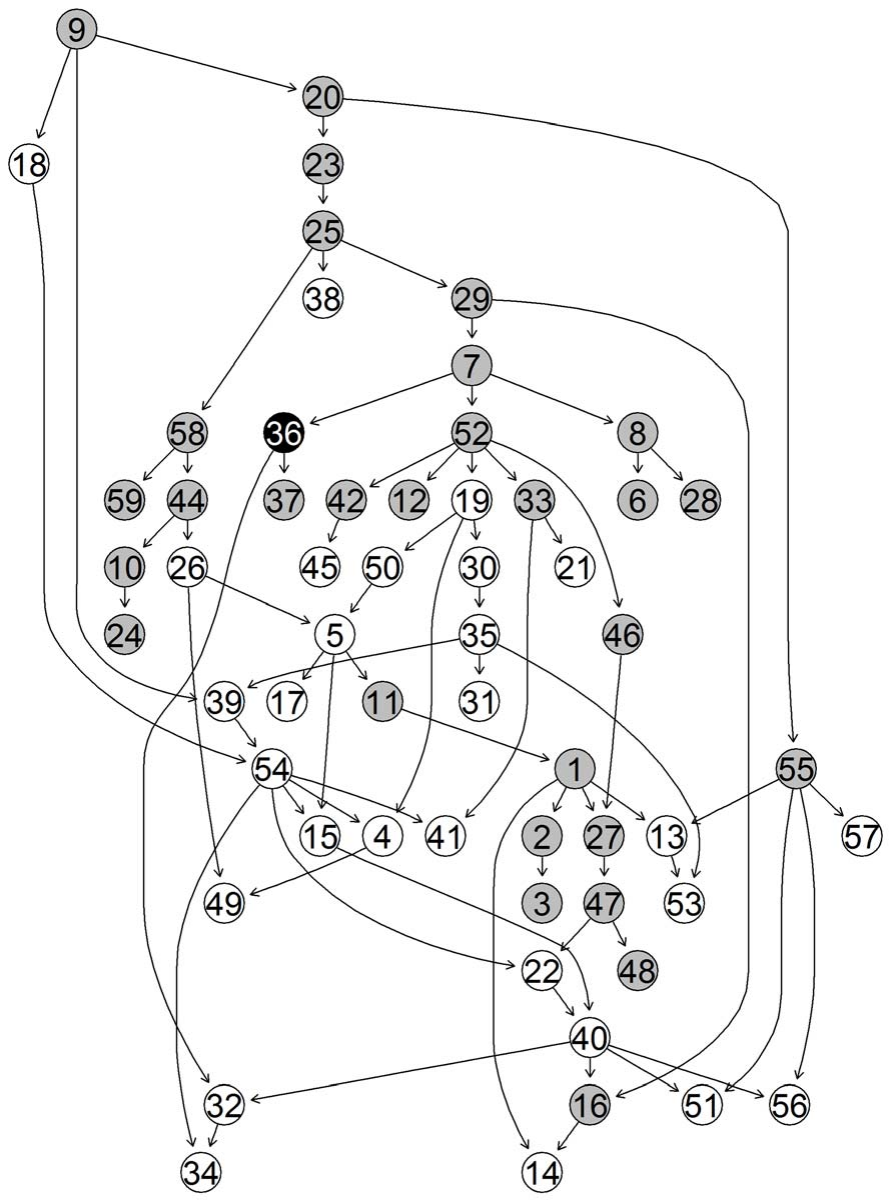
Network 2. Unique-Network for wheat under stress-enriched conditions in cluster 2. Grey nodes indicate highly predictive (average correct-prediction level higher or equal to 0.6) genes. Black nodes highlight highly predictive and stress related genes.

**Figure 11:**
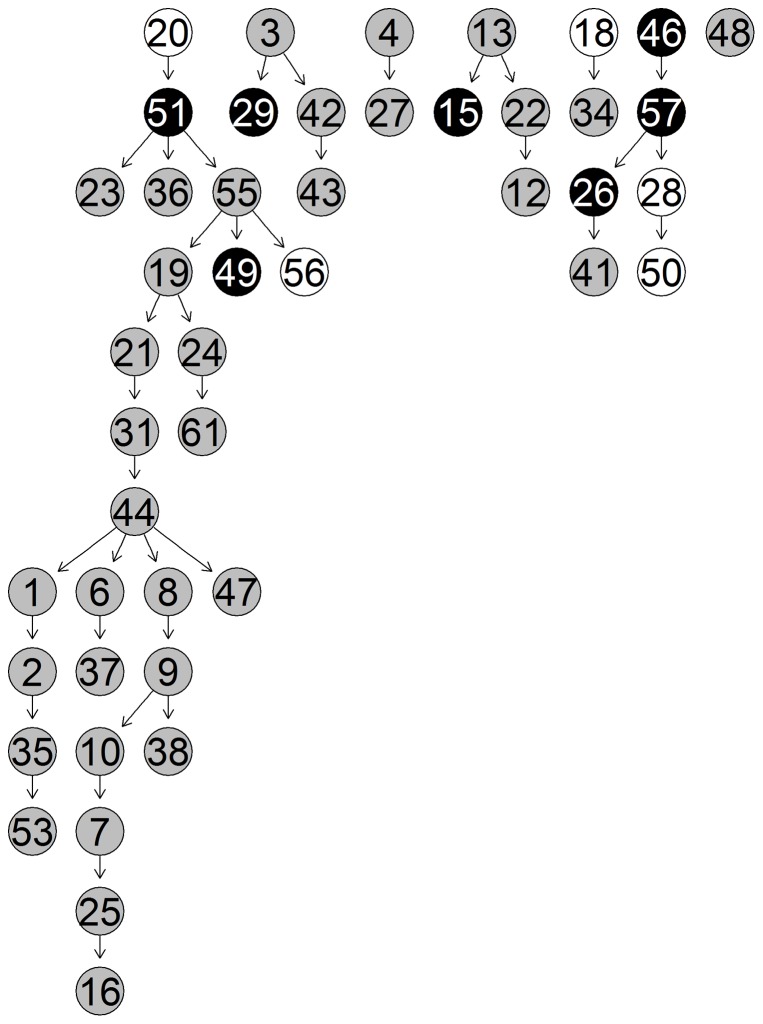
Network 3. Unique-Network for wheat under non-stress conditions in cluster 3.Grey nodes indicate highly predictive (average correct-prediction level higher or equal to 0.6) genes. Black nodes highlight highly predictive and stress related genes.

Once the relevant genes are selected, following the original step of our pipeline, we apply *glasso* to build a network for each study. We then calculate the sensitivity measure in order to cluster the studies based on graphical similarities. As for the simulated data, we explored k-means which generated the most convincing study-clusters. We explored different values of k but found that 3 clusters were the most revealing. Table 2 demonstrates that the studies can be grouped in two: stress-enriched and non-stress conditions. The resulting clusters are: 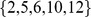, 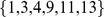 and 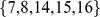 based upon the studies numbering from Table 2. While the third cluster clearly groups together all the non-stress studies, the other two reflect studies that are stress enriched. In the figures below we show the unique-networks, learnt with *bnlearn*, for wheat in the two study-clusters of stress-enriched conditions (Figures 9 and 10) and the unique network for the non-stress conditions cluster (Figure 11). The numbers identify the genes and the black circles represent in both the highly predictive genes that are involved in biotic (caused by living organisms) and abiotic (caused by non-alive factors) stress response. In both networks we clearly see specific paths and groups of genes that are highly connected. Using Mapman [21] we were able to associate a function to each gene.

Focusing on the stress-enriched conditions network, the procedure has managed to identify a relatively small number (58) of well-connected nodes which form a distinctive path. We see that genes involved in both kinds of stress response (biotic and abiotic stress) are involved in the network. Specifically the first four genes that start the network pathway in Figure 9 (29 – 47 – 17 – 30) are all involved in biotic stress. The remaining highlighted genes instead are mostly involved in heat stress. A good number of photosynthesis related genes are also involved, in particular (18 – 27 – 21 – 28 – 6 – 22 ). On the non-stress related network in Figure 11, we have again identified a reasonable number of genes though these are less connected. However, one very well defined pathway exists that consists mainly of photosynthesis-related genes (not highlighted).

**Figure 12:**
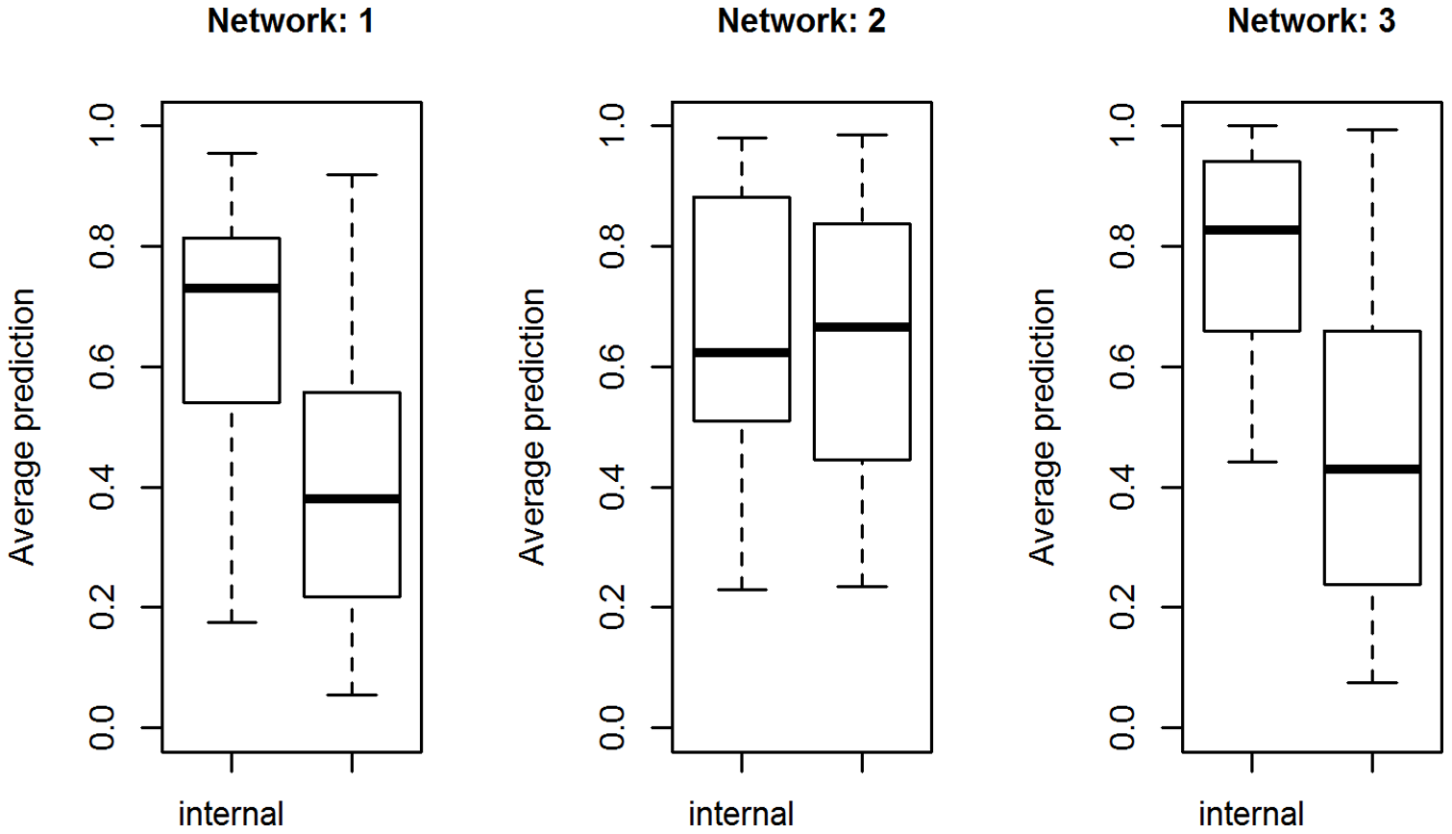
Boxplot intra vs inter clusters correct-prediction.

In the same network in Figure 11, less genes are found that are related to stress response and those that do appear are much less connected, except for the path formed by (46–57–26–50) nodes. The software described in [22] returns the following (see Table 3) highlighted biological functions which go to reinforce the results from Mapman. Higher values of Information Content (IC) are associated with more informative terms. Values greater than 3 are generally considered to be biologically informative. In the Figure 12 we show the predictive accuracy for each gene. What we expect is a better correct-prediction within the study-clusters and a weaker one outside the clusters. Each boxplot represents the percentage of how many times the gene has been predicted correctly among all the different given samples.

**Table 3:** Wheat Unique-Networks(U-N) biological process functions from Gene Ontology as described in [22].

U-N	GO Id	GO Name	IC
1	GO:0019538	protein metabolic process	3.19
1	GO:0006950	response to stress	3.96
1	GO:0071840	cellular component organization or biogenesis	3.98
2	GO:0006950	response to stress	3.96
2	GO:0071840	cellular component organization or biogenesis	3.98
2	GO:0019684	photosynthesis, light reaction	8.32
2	GO:0044267	cellular protein metabolic process	3.45
3	GO:0006950	response to stress	3.96
3	GO:0015979	photosynthesis	7.13
3	GO:0071840	cellular component organization or biogenesis	3.98
3	GO:0009628	response to abiotic stimulus	4.97
3	GO:0042221	response to chemical stimulus	4.12
3	GO:0006091	generation of precursor metabolites and energy	5.14
3	GO:0044267	cellular protein metabolic process	3.45

The chance of correctly predicting the genes randomly is one in three (there are three possible states for each gene: *under-regulated*, *normal*, *over-regulated*). Values above this can be considered better than random. In the figures we clearly see that the *intra cluster correct-predictions* (correct predictions made by cross validating within a study-cluster) are quite high for most of the genes with little variations. For the *inter clusters correct-predictions* (correct predictions on data outside of the study-cluster), however, the mean correct-prediction values are mostly not better than chance as one would expect, and the standard deviations are very high making them not reliable. In the majority of the cases, in fact, when a gene has an extremely high intra cluster correct-prediction it also shows a very low or a wide standard deviation in the inter clusters correct-prediction graph. This implies that the identified subnetworks are indeed specific to their study cluster, making them easier to characterise.

#### Comparison with Bicluster

Finally, we compare the results obtained with our algorithm in wheat with the one obtained using the Spectral Bicluster algorithm [31] in the R package *biclust* [32]. The method, after appropriately tuning the parameters, identifies 17 biclusters. On the wheat data each resulting bicluster highlights a different set of samples but the same set of six genes, 5 of which are related to abiotic heat stress. The genes highlighted by biclustering are also in the list of genes detected by the algorithm described in this paper, specifically we can see five of these genes also highlighted in Figure 9 (23 – 25 – 41 – 46 – 53). This discovery points out the importance of these 5 stress-related and 1 protein-degradation-related genes but unfortunately biclustering fails at identifying other equally important stress-related genes identified by our algorithm. In addition the six genes that are identified do not seem to be associated with a specific subset of samples. Rather each of them have been detected in all of the biclusters. Regarding the samples, about half of the biclusters manage to group together samples of stress-enriched studies but split samples from the same study. Unfortunately, none of the biclusters group the non-stress studies accurately enough to identify specific non-stress clusters. Furthermore, considering that each study consists of both actual treatment samples and a small number of controls it might be that biclustering merges together the control samples of the stress-conditions with non-stress samples but this union occurs too often and with too many samples for this to be considered the case. In conclusion, we have found that the resulting biclusters do not properly cluster the samples together, even ones belonging to the same study. Every bicluster highlights the same group of genes preventing any discovery of differences between treatments. It still discovers some important genes but much less than the ones we are able to find with the method proposed in this paper.

#### Comparison with WGCNA

As previously pointed out the *glasso* technique goes beyond simple pairwise relationships estimating a sparse inverse covariance matrix using the lasso (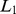) penalty. We compare it with the WGCNA (Weighted Gene Co-expression Network Analysis) technique as explained in section 0.6 of Materials and Methods. We applied both the scale free criterion for each study obtaining an array of different values of beta and then with only one value of beta set to 6 which is suggested to be the most appropriate value [41]. In both cases the results are extremely similar. Of the three clusters obtained with k-means only one of the stress clusters is quite reliable while the other two are quite mixed or meaningless (only two elements). Furthermore the unique networks reveals very small size graphs with much less nodes (less than 10) involved and very few connections. The small number of nodes detected in WGCNA have also been previously detected in *glasso*. As expected, the intra cluster correct-prediction is extremely good for the genes involved in each study-cluster, but, in this case, the number is so little that these results leave some strong doubts on the WGCNA algorithm usability on this dataset. Next, we show another case of real data analysis with Fusarium microarray data.

### Fusarium microarray Data

**Figure 13:**
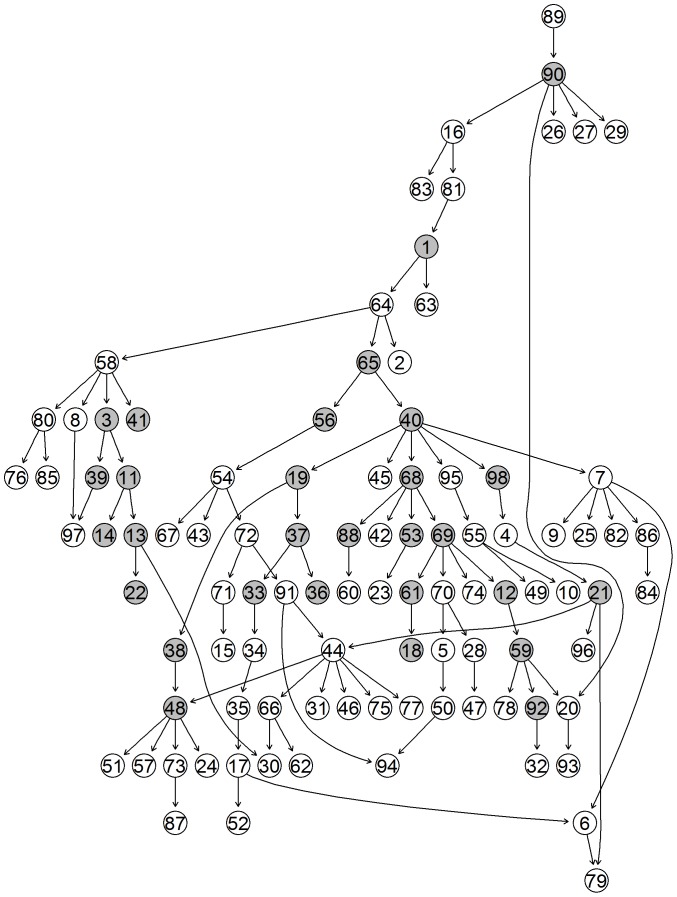
Unique-Network for *Fusarium* cluster 2,5,6,7,13. In this figure grey background indicates highly predictive genes (average correct-prediction equal or higher than 0.6). Despite the lack of different conditions in the dataset, as explained in the text, still about a 1/3 of the genes selected are highly predictive.

**Figure 14:**
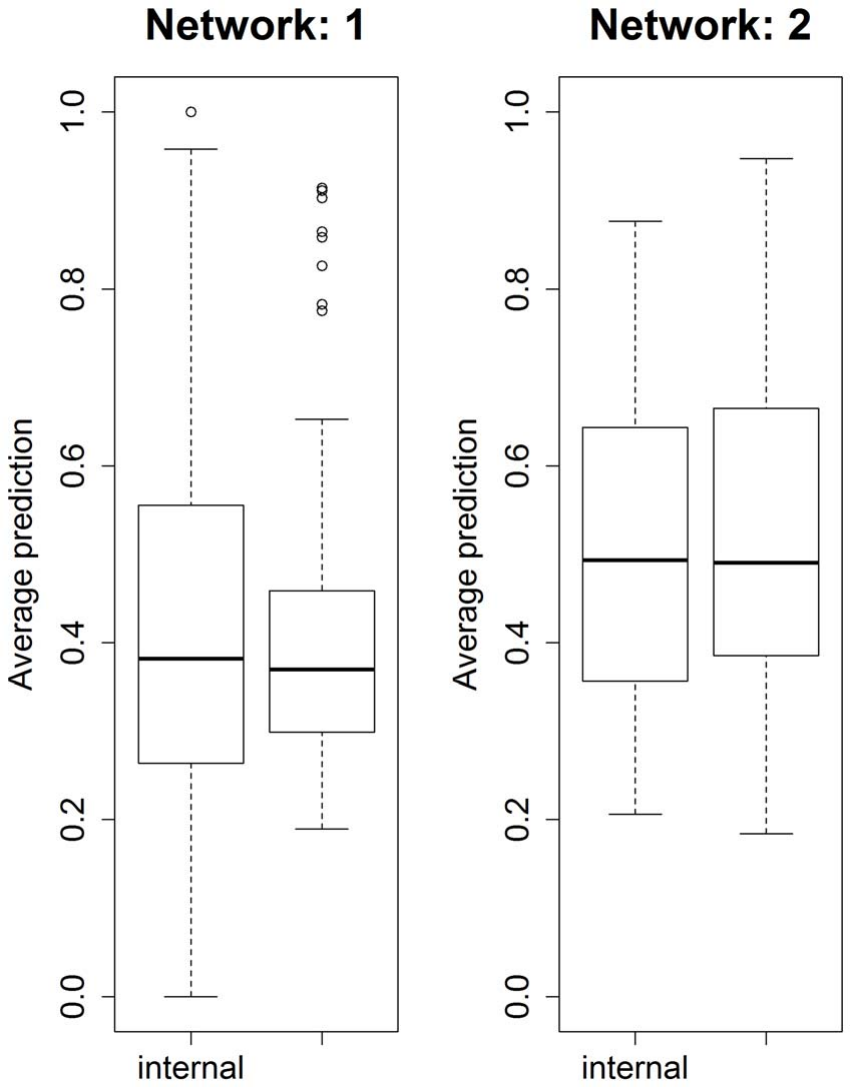
Intra vs inter clusters prediction for *Fusarium*.

Together with wheat, we also analyse a *Fusarium* graminearum dataset. The microarrays related to this organism (downloaded from [42]) include 18069 genes and 158 samples gathered in 13 treatments as shown in Table 4. We apply the variable selection, as described in Section 0.1 of Materials and Methods/pipeline, and we reduce the number of variables from 18069 to 98. Unlike in the wheat dataset, *Fusarium* studies are not easy to group at a first sight. What we decided to do then is to apply the *glasso* algorithm and calculate the sensitivity measure as it has been done before and then we apply k-means with different values of k and verify if there is any constant pattern. We repeatedly change the value of k in a range from 2 to 10 and we find that two groups of studies (of 5 and 2 studies respectively) always group together. This allows us to identify two study groups: cluster 1: 8,11 and cluster 2: 2,5,6,7,13. These studies do not belong to any stress condition, but they are recognized to have a similar underlying mechanism through the sensitivity measure. After the cluster detection we build the bayesian unique networks for these two groups. Because of their similarity here we show only the unique network for the second group in Figure 13. All 98 variables selected looks to be involved in both study-groups unique networks (except number 45 in the unique for cluster 1). This is because there are no major theoretical differences between the two study-group which means that the underlying mechanism might have only slight differences. The intra cluster correct-prediction shows for both clusters a very good correct-prediction accuracy. For the first cluster, because of its size (only 2 studies) we need to consider only genes with a very high accuracy average and a limited standard deviation range. Only few genes respect these criteria in both clusters. But a very limited number of genes results being very predictive in cluster one and not in cluster two and viceversa. The intra cluster correct-prediction for both groups is shown in Figure 14.

**Table 4:** Study numbers, labels, number of samples and descriptions of the *Fusarium* microarray dataset.

Fusarium Studies			
Study	Label	Number samples	Description
1	FG11-CEL	9	Gene Regulation by Fusarium TFs Tri6 and Tri10
2	FG13-CEL	18	The TF FgStuAp influences spore development,
			pathogenicity and secondary metabolism in Fusarium graminearum
3	FG14-CEL	8	DON induction media
4	FG2-CEL	9	Expression Profiles in Carbon and Nitrogen Starvation Conditions
5	FG3-CEL	14	Cross-species hybridization
6	FG1-CEL	18	Fusarium transcript detection on Morex barley spikes
7	FG12-CEL	15	Fusarium graminearum gene expression during crown rot of wheat
8	FG6-CEL	9	Transcript detection during in vitro sexual development of Fusarium
			Cch1 calcium channel deletion mutant
9	FG10-CEL	6	Response to trichodiene treatment in Fusarium graminearum
10	FG7-CEL	12	Fusarium gene expression profiles during conidia germination stages
11	FG16-CEL	12	Fusarium graminearum gene expression in wheat stems during infection
12	FG4-CEL	5	Fusarium/Barley RNA dilution
13	FG5-CEL	23	Fusarium transcript detection during in vitro sexual development

We now apply the algorithm in [22]. Since both networks involve the same genes they both have the same main functions. In Table 5 we show the main functions. Mapman was not applicable because it does not contain *Fusarium* data.

**Table 5:** *Fusarium* unique networks biological process functions from Gene Ontology as described in [22].

GO Id	GO Name	IC Term
GO:0004175	endopeptidase activity	6,93
GO:0015179	L-amino acid transmembrane transporter activity	6,90
GO:0004497	monooxygenase activity	6,23
GO:0008324	cation transmembrane transporter activity	6,16
GO:0005506	iron ion binding	6,12
GO:0022804	active transmembrane transporter activity	5,29
GO:0022891	substrate-specific transmembrane transporter activity	3,78
GO:0046872	metal ion binding	3,50
GO:0016491	oxidoreductase activity	3,15

These results show us that even if the clusters have a similar underlying mechanism we still can identify few genes that are highly predictive and therefore characteristic of the clusters. These results can be compared to the one found for the simulated data with a higher level of noise.

#### Comparison with WGCNA

At this point we explore the WGCNA technique and compare it with *glasso*. As explained in *Materials and Methods* we first calculate the co-expression similarity matrix and convert it into the adjacency matrix using the scale-free topology criterion. Here again the clusters are organized differently and are not as significant as the ones obtained with *glasso*. The unique networks include far fewer genes and the internal correct-prediction also shows less highly predictive genes compared to the ones we found using *glasso*.

Based on the poor results previously obtained from applying biclustering, we decide not to apply this technique on this dataset.

## Discussion

A key focus of this paper is the exploration of wheat of which there is still much uncertainty. We now explore in some detail the biological feedback based on the discovered unique networks. The three networks in Figures 8–10 are indicative for different sample sets e.g different stress conditions. They represent increase in the gene transcription for certain genes and the links between them. Eighty percent of Networks 1, 2 and 3 are consistent with the literature. The remaining twenty percent did not present direct correlation though there is evidence for some correlation in database sources such as [43]–[45]. First, the main genes correlated to biotic stress were basic chitinase. Basic chitinases are antimicrobial proteins that are capable of degrading fungal cell wall chitin. They are two classes either basic or acidic isoelectric points [46]. Gene 19 (PR3 (Basic chitinase)) in network 2 (NW2) in Figure 10 (30 in NW1, Figure 9; 15 in NW3, Figure 11) is correlated to gene 30 (allergen V5/Tpx-1-related family protein) in NW2, followed by 35 (BMY1, (BETA-AMYLASE)) in NW2 and 31 (PR3, (Basic chitinase)) in NW2. Basic chitinase (19 in NW2) also affects 49 (CK215257 Dirigent-like superfamily) via gene 4 (cysteine proteinase, putative). Allergen V5, pathogenesis related 4 and basic chitinase (29, 47, 17 in NW1; 30, 19 and 50 in NW2, respectively) are represented in both networks with different links between the gene expressions. Differently in network three (NW3) begins with gene 20 (PR3, (Basic chitinase)) followed by 51 (HEL, PR-4, (Pathogenesis-related 4)) and 36 (BMY1, (Beta-amylase)), where allergen V5 is completely missing. Therefore we conclude that gene expression of allergen V5 may be only visible under certain stress conditions.

Glycine decarboxylase complex H (gene 39, NW1) was correlated to transcription of Rubisco gene (56, NW1) that regulated genome uncouples 5 (GUN5). GUN5 is a plastid derived signal that plays an important role in the coordinated expression of both nuclear and chloroplast localised genes that encode photosynthetic-related proteins [47]. It regulated genes 21 (LHCA1), 28 (PSAK (Photosystem subunit K)), 6 (LHCB5 (Light harvesting complex of photosystem II 5), 22 (PSAD-1 (photosystem I subunit D-1)) and 4 (cysteine proteinase, putative) and gene 18 (LHCB1.5, Photosystem II light harvesting complex gene 1.5). Followed by gene 27 (LHCB3*1, Light-harvesting chlorophyll binding protein 3) and 5 (RNS1 (Ribonuclease 1); endoribonuclease) confirming its functional properties. In NW2 the relationship between Rubisco (gene 58, NW2) and glycine decarboxylase complex H (44, NW2) seems to be in the opposite direction. The previously published data suggest that the expression of both genes is light dependent and tissue specific, which is due to 259-bp upstream region of the promoter region [48]. In both NWs ferredoxin gene (59, NW2) and (57, NW1)) was linked to Rubisco and glycine decarboxilase complex. Due to physiological importance of these genes in both networks the two relationships could be correct. In NW3 the photosynthetic reaction is regulated by MYB like transcription factor (19, NW3) and glycine decarboxylase complex (44, NW3) while the transcription of Rubisco gene is below the level of significance [49].

Photosystem I was represented by genes 22 and 28 in NW1; 24, 29 and 22 in NW2; and 24, 31 34 in NW3. The photosystem I composed of four complex (Lhc (light harvest complex) proteins and a1-Lhca4 belonging to the light harvesting protein family [50]. Also the light harvesting complex II (LHCII) is implicated by the regulation of excitation energy distribution between Photosystem I (PSI) (21, NW 1) and Photosystem II (PSII) (6, NW 1) during the state transition and also light-harvesting complex II binds to several small subunits of photosystem I [51]. PSI-K subunit of photosystem I (28, NW1; 29, NW2 and 31, NW3), is involved in the interaction between light harvesting complex I and the photosystem reaction centre core [52], [53].

The main trimeric light-harvesting complex of higher plants (LHCII) consists of three different Lhcb proteins (Lhcb 1-3) in Arabidopsis thaliana. In NW1 these genes are 27 (LHCB3*1, (Light-harvesting chlorophyll binding protein 3) and gene 18 (LHCB1.5, (Photosystem II light harvesting complex gene 1.5)) [54]. Gene 6 or LHCB5, (Light harvesting complex of photosystem II 5), this gene is significant because is affected by different light regimes in rye plants. It may be also indicative for wheat function due to the high similarity in the gene sequences between wheat and rye. In NW2, the genes 7, 8 were the same as in the NW1. Also gene 33 (PSAN (photosystem I reaction centre subunit PSI-N); calmodulin binding), 42 (APX4 (Ascorbate peroxidase 4); peroxidase) are related due to their function in photosynthesis [55].

Other fundametal parts of the network are the group of heat shock proteins. The major groups are HSP100, HSP90, HSP70 and they are also confirmed in wheat [56]. The novel finding in NW1 is that the genes indicated by 41 (HSP70), 23 (HSP101 (Heat Shock Protein 101)), 53 (HSP70), 25 (HSP21) and 46 (ATHP22.0) are related to a protein degradation gene 54 (CLPP_wheat.gb/CA607537) which is 98% similar to AB042240 Triticum aestivum chloroplast (http://www.ncbi.nlm.nih.gov/nucleotide/13928184). This finding provides new insights into relationships between heat shock proteins and this particular chloroplast gene that seems to have a regulatory function over the sequence in Figure 9. In NW2 transcripts for heat shock proteins were not present.

In NW2 the main effects were indicated with the gens MLP-like protein (39, NW2 and 35, NW1), beta amylase (35 in NW2 and 33 in NW1) and rare-cold inducible (RCI) 54, NW2 and 51, NW1). The MLP-like protein is related to beta amylase but there was no explanation exactly how [57]. The link with rare-cold inducible protein and one helix protein seems impossible because rare cold inducible protein is expressed in the roots and is mainly restricted to endodermis [58], one helix protein belong to one of the light-harvesting chlorophyll a/b-binding (Lhc) proteins [59]. More research would be required to prove or disprove the relationship between them. Transcript for MLP-like protein in NW3 was not detected to be involved in the network (Figure 11; NW3).

ATPRX Q; antioxidant gene (42, NW1 and 46, NW2 and 47, NW3) is central for NW1 and NW2 but peripheral for NW3. It is highly expressed in leaves and low expressed in the stem. Its expression patterns indicated that is induced by ultraviolet irradiation, low temperature and salt stress. The induction of Prx in response to abiotic stimuli may suggest that Prx may protect the host against environmental stresses [60]. It looks like gene 42 affects gene 41 (HSP70T-2; ATP binding) and gene 7 (PSBS, (Nonphotochemical quenching), 16 (lipase, putative) and 38 (APX4 (Ascorbate peroxidase 4); peroxidase) and it is itself affected by 39 (GDCH (Glycine decarboxylase complex H)).

The transcript of the chloroplast glyceraldehyde-3-phopshate dehydrogenase (phosphorylating, E.C 1.2.1.14) (GADPH) (38 (GAPA-2–GAPA-2) was only found in NW2. In higher plants exists as heterotetrameter that catalyses the reductive step of the Calvin cycle [61]. GAPA-A subunit was also identified chloroplast localized proteins [62]. GAPDH is a classical glycolytic enzyme that is involved in cellular energy production and has suppressed heat shock-induced peroxide production and cell death [63]. It is also involved in spontaneous assembly of photosynthetic supramolecular complex with CP12 protein that contributes to Calvin cycle regulation and phosphoribulokinase (PRK) in photosynthetic organisms [64]. It is surprising that the tree proteins GAPDH, CP12 and PRK are not expressed together [65]. The importance of this gene is its involvement in photosynthesis and Calvin cycle regulation at the same time. Its strategic place in our NW2 points that this gene could be a potential target for further investigation to establish the relationships and regulatory function in both processes.

Based on these biological findings we can conclude that our pipeline is a robust and reliable method to analyse large sets of transcriptomic data. It easily detects the main complex relationships between transcriptional expression of genes specific for different conditions and also highlights structures and nodes that could be potential targets for further research.
